# The Moderation Role of Acculturation on Dietary Patterns and Stress among U.S. Chinese Immigrants

**DOI:** 10.1093/geroni/igab046.3280

**Published:** 2021-12-17

**Authors:** XinQi Dong, Qun Le

**Affiliations:** 1 Rutgers University, Rutgers Institute for Health, New Jersey, United States; 2 Rutgers University, New Brunswick, New Jersey, United States

## Abstract

Studies have demonstrated that healthy dietary patterns are related to diminished stress. However, the potential moderation role of acculturation on dietary patterns and stress is unclear among the those whose eating habits are impacted by immigration. The aim of this study is to explore the moderation role of acculturation on dietary patterns and stress among Chinese elder immigrants in the United States. Data were conducted from the PINE Study with 3053 Chinese adults aged over 60 years in the Great Chicago area. Dietary patterns were measured via a 48-items Food-frequency questionnaire with frequency and size weighted. Items were identified into different food groups based on Dietary Guidelines. Acculturation was assessed by a 12-item short-scale among the population. Stress was measured via a 10-item Perceived Stress Scale with cutoff 14 indicating either low or high stress. Multiple logistic regression was used to examine the moderation effects on the associations with demographic characteristics, medical comorbidities, and BMI adjusted. After controlling covariates, one unit increasing in fruit consumption (OR: 0.61 (95%CI: 0.52 -0.72)) or coffee (OR: 0.49 (95%CI: 0.36 - 0.67)) was associated with lower odds of stress. However, after adding acculturation as an interaction term, the negative relationship between fruit or coffee consumption with stress was moderated by a higher level of acculturation (fruit: OR: 1.05 (95%CI: 1.02 - 1.08), coffee: OR: 1.05 (95%CI: 1.01 - 1.09) respectively). The associations between dietary patterns and stress may differentiate based on acculturation level among the elder immigrants. Further longitudinal studies should investigate potential causality.

